# Dry spells trigger durian flowering in aseasonal tropics

**DOI:** 10.1007/s00484-024-02819-x

**Published:** 2024-11-12

**Authors:** Aoi Eguchi, Noordyana Hassan, Shinya Numata

**Affiliations:** 1 Graduate School of Urban Environmental Sciences, Tokyo Metropolitan University, Minami- Osawa 1-1, Hachioji, Tokyo, Japan; 2 Geoscience and Digital Earth Centre (INSTeG), Research Institute of Sustainable Environment, Universiti Teknologi Malaysia, Skudai, Johor Malaysia; 3 Department of Geoinformatics, Faculty of Built Environment and Surveying, Universiti Teknologi Malaysia, Skudai, Johor Malaysia

**Keywords:** Drought, Environmental response, First flowering date, Phenology, Tropical fruit

## Abstract

The flowering period of durian is influenced by weather conditions such as prolonged drought and low temperatures. However, the specific criteria and durations of these conditions remain unclear. This study aimed to identify weather conditions that trigger durian flowering by monitoring its phenology and analyzing the relationship between minimum temperature and rainfall using two types of durian— grafting type and seed-grown type. Three flowering events were observed in 2022 and two in 2023. Considering there were no differences in the number or timing of flowering events among the two types, it suggests that genetic background differences do not influence flowering phenology. All flowering events were best explained using the 15-day moving average of rainfall, with the peak of the first flowering occurring approximately 50 days after the 15-day moving average of rainfall fell below 1 mm. These results suggest that drought accumulation over approximately 15 days may trigger the induction of durian flower buds. However, no correlation was determined between the minimum temperature and the first flowering date. These findings indicate that durian flowering is induced by milder drought conditions than those previously proposed. Differences in variety and climate may have led to results different from those reported in earlier studies. Long-term monitoring across multiple sites and varieties is required to discuss these differences further.

**Supplementary Information:**

The online version contains supplementary material available at 10.1007/s00484-024-02819-x.

## Introduction

The timing of seasonal events in plants, such as flowering, is highly sensitive to climate change and is widely used as an indicator to assess its effects (Fitter and Fitter 2002; Cleland et al. 2007). The reproductive season in tropical rainforests in Southeast Asia is particularly vulnerable to climate change (Numata et al. 2022). The proposed environmental cues for flowering and fruiting in tropical rainforests include temperature, rainfall, and solar radiation (Ashton et al. 1988; Borchert et al. 2005; Chen et al. 2018). However, the exact mechanisms or specific triggers of flowering and fruiting in tropical climates with low seasonality remain unknown. Despite its importance, plant phenology research in the tropics is limited, due to the lack of long-term studies (Abernethy et al. 2018).

Durian (*Durio zibethinus* L.), the “king of fruits,” is an evergreen tree native to Southeast Asia (Subhadrabandhu and Ketsa 2001). Durian is an economically valuable fruit that accounts for the largest share of all Malaysian fruits in terms of production, area planted, and quantity produced (Department of Agriculture Malaysia 2022). It is, therefore, economically important to predict future changes in durian production. Durian is harvested from May to August and from November to December in Peninsular Malaysia (Lim and Luders 1997). It has been reported that the timing of durian flowering is affected by drought and low temperatures that last for a certain period (Chandraparnik et al. 1992; Salafsky 1995; Lim and Luders 1998; Zainal and ZainalAbidin 2002; Salakpetch 2005). Zainal and ZainalAbidin (2002) reported that drought with rainfall < 1 mm/d for a continuous 18-day period triggers floral initiation. In terms of low temperatures, Lim and Luders (1998) reported that several days of cold nights below 15°C in Australia triggered floral initiation after 4–6 weeks. However, other studies did not provide specific values or durations that define drought or low temperatures. Although it is widely believed that drought and low temperatures empirically affect the flowering period of durians, this has not been precisely tested.

The genus *Durio* is native to Southeast Asia, and two species (*Durio dulcis* and *Durio oxleyanus*) are sympatric with general flowering, which occurs every few years in Southeast Asia (Fredriksson et al. 2006). General flowering is a phenomenon in which various plant taxa bloom simultaneously, indicating that these plants may respond to similar weather cues for flowering. General flowering may be triggered by the accumulation of synergistic effects of drought and low temperatures for 54–90 days (Chen et al. 2018). The timing of life history events is covarying with the phylogeny, and it has been found that more closely related species tend to have flowering periods that occur at the same time (Davies et al. 2013). It is, therefore, highly likely that similar weather cues are used for the flowering of durian owing to its close relationship with two *Durio* species that synchronize with general flowering.

Previous studies have suggested that weather conditions such as drought and low temperatures are likely involved in the flowering of durian (Chandraparnik et al. 1992; Salafsky 1995; Lim and Luders 1998; Zainal and ZainalAbidin 2002; Salakpetch 2005). However, these studies rely on anecdotal reports without clearly identifying their sources, and the definitions of drought and low temperature are either missing or ambiguous. Therefore, in this study, the phenology of durian flowering was monitored and the relationship between rainfall and minimum temperature was analyzed to identify the specific weather conditions that trigger flowering in durian. Based on previous studies, the hypothesis of specific periods of drought lasting two weeks to two months and the accumulation of low minimum temperatures preceding flowering was proposed and tested.

## Materials and methods

### Study area

The survey was conducted in an orchard of the University Technology Malaysia (1.56°N, 103.62°E), located in Johor, southern Peninsular Malaysia. Peninsular Malaysia has a tropical rainforest climate characterized by high temperatures and humidity throughout the year. The annual average temperature ranges from 25 to 32 °C, and the annual rainfall is between 2000 and 4000 mm (Suhaila and Jemain 2007). Rainfall patterns in Johor are influenced by monsoons. The northeast monsoon from November to February results in increased rainfall, while the southwest monsoon from May to August is a relatively dry period; heavy rainfall can occur as convective rain during the two inter-monsoon seasons (Suhaila et al. 2010).

### Durian phenology monitoring

The durian trees (*D. zibethinus*) observed in this study consist of two types: grafted and seed-grown. The grafted trees belong to established varieties selected for desirable traits and propagated by grafting, which minimizes genetic variation. In total, 150 varieties have been registered with the Department of Agriculture Malaysia (Department of Agriculture Malaysia 2023). In contrast, the seed-grown trees were cultivated from seeds, which are referred to as “Durian kampung” in Malaysia. The study covered 110 durian trees, half of which were the grafting type, and the other half were seed-grown type, all of which were approximately 20–30 years old. Owing to the loss of information during the handover between farm managers, the number and names of the cultivars for grafting-type trees, as well as the origin and identity of the seed-grown trees, are unknown.

The day on which the first flowering was observed was recorded as the first flowering date (FFD) for 110 durian trees. The flowering was observed using binoculars. A flowering event was defined as the occurrence of five or more observed trees flowering simultaneously. The first day of the flowering event was defined as the earliest FFD among the trees that participated in the event. The event was considered to have ended when no flowering activity was observed for at least one week. The flowering phenology of all the target trees was monitored from January 3, 2023, to April 18, 2024. Flowering was consistently observed every other day during the flowering seasons (January 15–March 17, 2023; April 27–May 29, 2023; and February 13–April 18, 2024). Observations were conducted only twice (August 29 and September 19) from July 7 to November 26, 2023. During the other observation periods (January 3–14, 2023; March 18–April 26, 2023; May 30–July 6, 2023; and November 27, 2023–February 12, 2024), observations were made at least once every two weeks.

### Meteorological data

Daily rainfall and temperature data from Senai, Johor (1.38°N, 103.40°E), located approximately 10 km from the survey point, were used in this study. Weather data were obtained from the Malaysian Meteorological Department for the period from December 1, 2022, to March 10, 2024.

### Data analysis

Owing to the methods of propagation, the seed-grown type is assumed to be genetically diverse, while the grafting type is genetically uniform. Given their differing genetic backgrounds, the flowering periods of these two types may vary. To clarify whether the flowering periods differed between reproductive types, the Mann-Whitney U test was used to examine the difference in the median FFD for each flowering event. The FFD were converted to the number of days elapsed from the first observed FFD of each event, which was counted as day 1. The analysis was conducted on flowering events 1–4, which lasted for two or more days, however, flowering event 5, which lasted only one day, was excluded from the analysis.

To evaluate prolonged drought, we calculated the five-day, 10-day, 15-day, 20-day, and 30-day moving averages of rainfall. Drought periods were defined as the periods when the moving average of rainfall was less than 1 mm. The number of drought periods during the study period was counted and compared to the frequency of flowering events.

## Results

### Flowering events

**Fig. 1:**
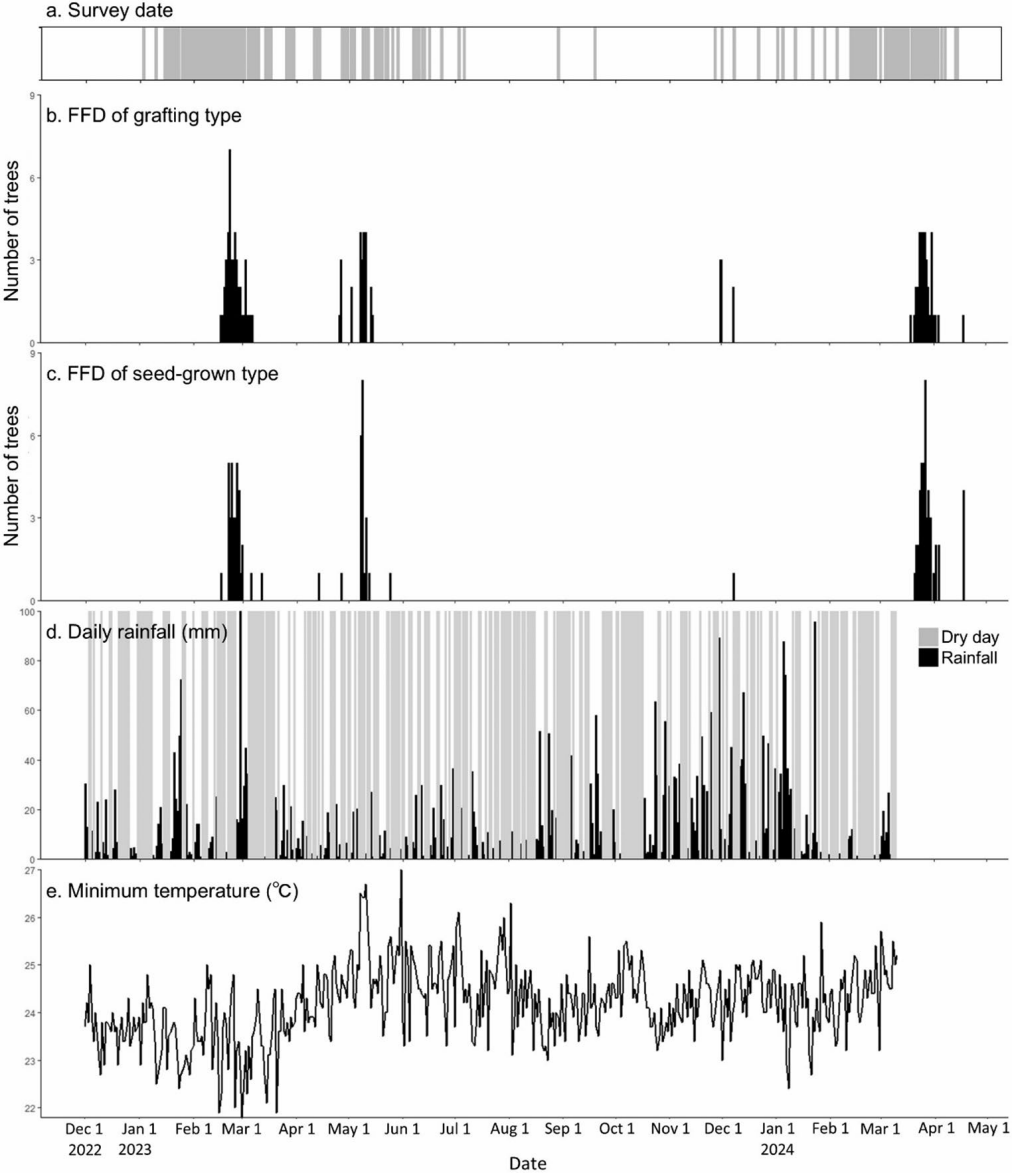
FFD of individual durian trees and weather data. (**a**) Survey date (gray color), (**b**) FFD of grafting type, (**c**) FFD of seed-grown type, (**d**) Daily rainfall, (**e**) Daily minimum temperature

A total of five flowering events were observed during the study period—the first from February 17 to March 12, 2023, the second from April 26 to May 15, 2023, the third from December 1 to 8, 2023, the fourth from March 19 to April 4, 2024, and the fifth from April 18, 2024 (Fig. 1). In addition, one tree flowering was observed on April 14 and one on May 25, 2023, but they were not counted as flowering events in this study because of their small number and distance of more than one week from other flowering events. Of the three flowering periods observed in 2023, harvest was observed only after the first flowering period. The number of individuals observed was 78 (grafting type: 44, seed-grown type: 34) in the first flowering period, with a peak of 10 on February 22, 2023; 44 (grafting type: 24, seed-grown type: 20) in the second, with a peak of 11 on May 9, 2023; six (grafting type: five, seed-grown type: one) in the third, with a peak of three on December 1, 2023; 77 (grafting type: 35, seed-grown type: 42) in the fourth, with a peak of 12 on March 27, 2024; and five (grafting type: one, seed-grown type: four) in the fifth, all bloomed on April 18, 2024. The results of the Mann–Whitney U test presented no significant difference in the median FFD for flowering events 1–4 (p-value > 0.05).

### Meteorological conditions

The mean temperature during the study period was 27.2 °C and the annual rainfall in 2023 was 3018.6 mm. Daily rainfall was relatively high from November to February and relatively low from March to August (Fig. 1). Consecutive dry days (CDD, where rainfall ≤ 1 mm) ranging from a minimum of four days to a maximum of 15 days were observed before each flowering event. The temperatures were relatively low from December to February and high around May and October; however, there was no decrease in minimum temperatures observed before flowering (Fig. 1).

### Prolonged drought before flowering

**Fig. 2:**
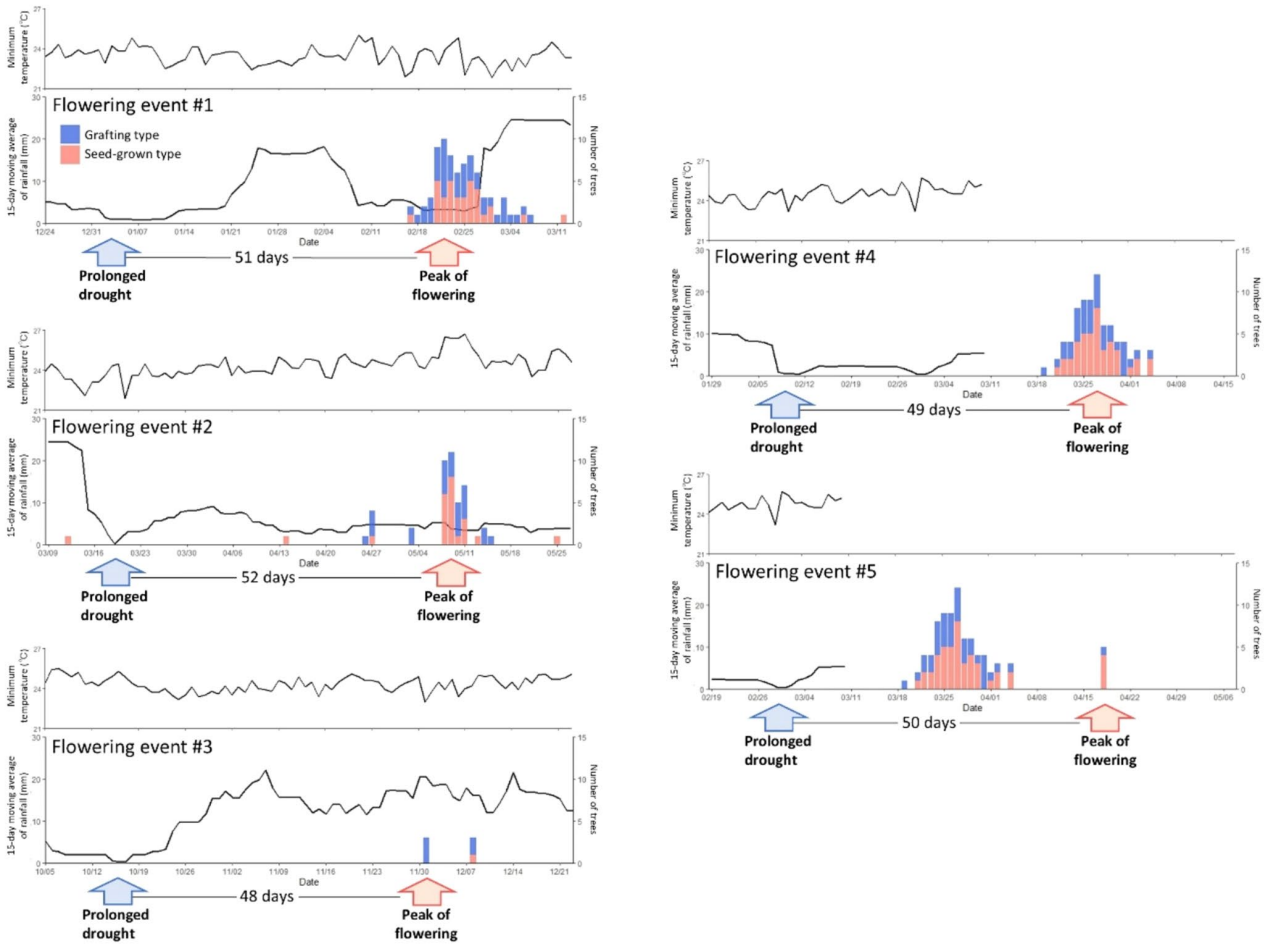
Relationship between FFD of individual durian trees, the 15-day moving average of rainfall, and the minimum temperature. Blue indicates the seed-grown type, and red indicates the grafting type

The five- to 30-day moving average of rainfall was calculated as an indicator of prolonged drought (Figure S1) and found that 15-day moving averages of 1 mm or less were observed before all the flowering events (Fig. 2). During the study period, the five-day moving average of rainfall fell below 1 mm 21 times, the 10-day moving average fell below 1 mm eight times, the 15-day moving average fell below 1 mm five times, the 20-day moving average fell below 1 mm one time, and the 30-day moving average never fell below 1 mm. When compared to the five flowering events observed during the study period, droughts lasting around five and 10 days occurred 4.2 times and 1.6 times more frequently, respectively. In contrast, droughts of approximately 20 days could only explain 20% of the five flowering events, as only one such drought occurred, and no 30-day droughts were observed. However, droughts lasting around 15 days were always observed before each of the five flowering events. The five dry periods are as follows: once for nine days from January 3 to 11, 2023; second for one day on March 19, 2023; third for three days from October 15 to 17, 2023; fourth for five days from February 8 to 12, 2024; and fifth for three days from February 29 to March 2, 2024 (Fig. 2). From the first day, the peak FFD was approximately 50 d when a 15-day rainfall moving average of 1 mm or less was observed (51, 52, 48, 49, and 50 d) (Fig. 2). However, there was no clear relationship between the date of the FFD and the maximum and mean temperatures or the moving average of other days (5, 10, 20, and 30 d; Figure S1).

## Discussion

The date of the peak flowering was best explained when the 15-day rainfall moving average was used, with the peak flowering observed approximately 50 days after the 15-day rainfall moving average fell below 1 mm (Fig. 2). This result suggests that the accumulation of dry conditions over approximately 15 days triggers the induction of durian flower buds. As the number and timing of flowering days were the same for both grafting and seed-grown types, it suggests that genetic background differences do not influence flowering phenology of durian. The previously reported flower bud induction requires 18 consecutive days with less than 1 mm of rainfall (Zainal and ZainalAbidin 2002). In the present study, however, the longest dry spell during the study period was 15 days, from March 5 to March 19, 2023, and none of the 18 consecutive days that previous studies have shown to be necessary for flowering was observed. In addition, the shortest CDD before flowering was four days, from 28 to 31 January and from 8 to 11 February 2024. Flower buds are, therefore, induced even during dry periods and occasional rainfall. This result suggests that, while dry conditions are necessary for flower bud induction, the stringent requirement of CDD discussed in previous studies may not be essential. The shortest CDD observed before flowering was four days, which occurred 22 times during the study period and 4.4 times more frequently than the number of flowering events. Thus, when discussing triggers for durian flowering, the moving average rainfall may be a more appropriate indicator of dryness than the CDD count.

The number of flowering individuals observed in the five flowering events may be related to the duration of the drought (15-day moving average of rainfall below 1 mm) and the resources within the individuals. In particular, during flowering events 1 and 4, the drought lasted for more than five days where a higher number of flowering individuals were observed. In other flowering events, the drought lasted for one to three days. As more severe droughts can trigger more flowering individuals (Vogado et al. 2023), it is believed that the number of flowering individuals increases. However, the number of flowering individuals may also be influenced by accumulated resources, in addition to the strength of the trigger (Sakai et al. 2006). Flowering events with a particularly high number of individuals were observed alternately from the other events. The effect of drought duration and intensity on the scale of flowering and fruiting remains a subject for future research.

Environmental triggers of durian flowering may be similar to those of general flowering in Southeast Asia. Among the 95 dipterocarp species, 34 dipterocarp species respond to both drought and low temperature cues for flowering (Numata et al. 2022), and those species responded to 54–90 days of accumulation of drought and low temperature (under thresholds determined by the model) (Chen et al. 2018). This suggests that durians may have shorter signal accumulation periods during drought and higher flowering frequency than wild trees in tropical rainforests. Furthermore, Chen et al. (2018) reported that the flower development period after the trigger ranged from 43 to 96 days depending on the species, which is consistent with the approximately 50 days observed in this study.

On the other hand, low temperature is a known trigger for general flowering and has been hypothesized as a possible flowering trigger for durians; however, the results of this study do not support this hypothesis. After four years of monitoring in Darwin, Australia, flower buds may appear after one to two weeks and flowering after four to six weeks when there are several days of cold nights below 15 °C (Lim and Luders 1998). However, in this study, no temperatures below 20 °C were observed during the study period (Fig. 2). It may, therefore, be concluded that drought may play a more important role than temperature in durian flowering in Peninsular Malaysia. The fact that the flowering triggers for general flowering are both low temperatures and drought, while durian flowering is triggered only by drought, may be a potential reason for the higher flowering frequency of durian compared to general flowering.

The harvesting season and durian frequency generally vary by region. Peninsular Malaysia typically has two seasons—May–August and November–December (Lim and Luders 1997). However, in the present study, flowering occurred three times in 2023. To the best of our knowledge, this is the first record of mature trees flowering three times a year and it represents a fundamental and significant record in terms of the phenology of durian. There are very limited records of durian flowering, except for one record of a young durian tree flowering three times a year in Peninsular Malaysia in 1975 (Yaacob et al. 1978). Only one of the three flowering events per year led to harvest, while the others did not. It may be that flowering, unrelated to harvest, has not been the focus so far because of its lack of agricultural productivity. Further phenological monitoring and flowering surveys at other sites are required to clarify whether this is a common phenomenon.

## Conclusions

All the flowering events were best explained using the 15-day moving average of rainfall, with peak flowering occurring approximately 50 day after the 15-day moving average of rainfall fell below 1 mm. These results suggest that drought accumulation over approximately 15 days may trigger the induction of durian flower buds. However, no correlation was determined between the minimum temperature and the first flowering date. These findings indicate that durian flowering is induced by milder drought conditions than those previously proposed. Differences in variety and climate may have led to results different from those reported in earlier studies. Long-term monitoring across multiple sites and varieties is required to discuss these differences further.

## Electronic supplementary material

Below is the link to the electronic supplementary material.

Supplementary Material 1

## Data Availability

The datasets generated during and/or analyzed during the current study are available from the corresponding author on reasonable request.
