# Supplementary material for: Dry spells trigger durian flowering in aseasonal tropics
Source: Int J Biometeorol. 2024 Nov 12;69(2):403–9. doi: 10.1007/s00484-024-02819-x (PMC11785592; doi:10.1007/s00484-024-02819-x)
Supplement: Supplementary file 1 — Supplementary Material 1 [file 484_2024_2819_MOESM1_ESM.pdf]

Supplementary Information

Title: What triggers durian flowering in the aseasonal tropics?

Journal: International Journal of Biometeorology

Authors: Aoi EGUCHI, Noordyana HASSAN, Shinya NUMATA

Corresponding author: Aoi EGUCHI

Affiliation: Graduate School of Urban Environmental Sciences, Tokyo Metropolitan University, Minami-Osawa 1-1, Hachioji, Tokyo 192-0397, Japan

E-mail: aoi82e@gmail.com

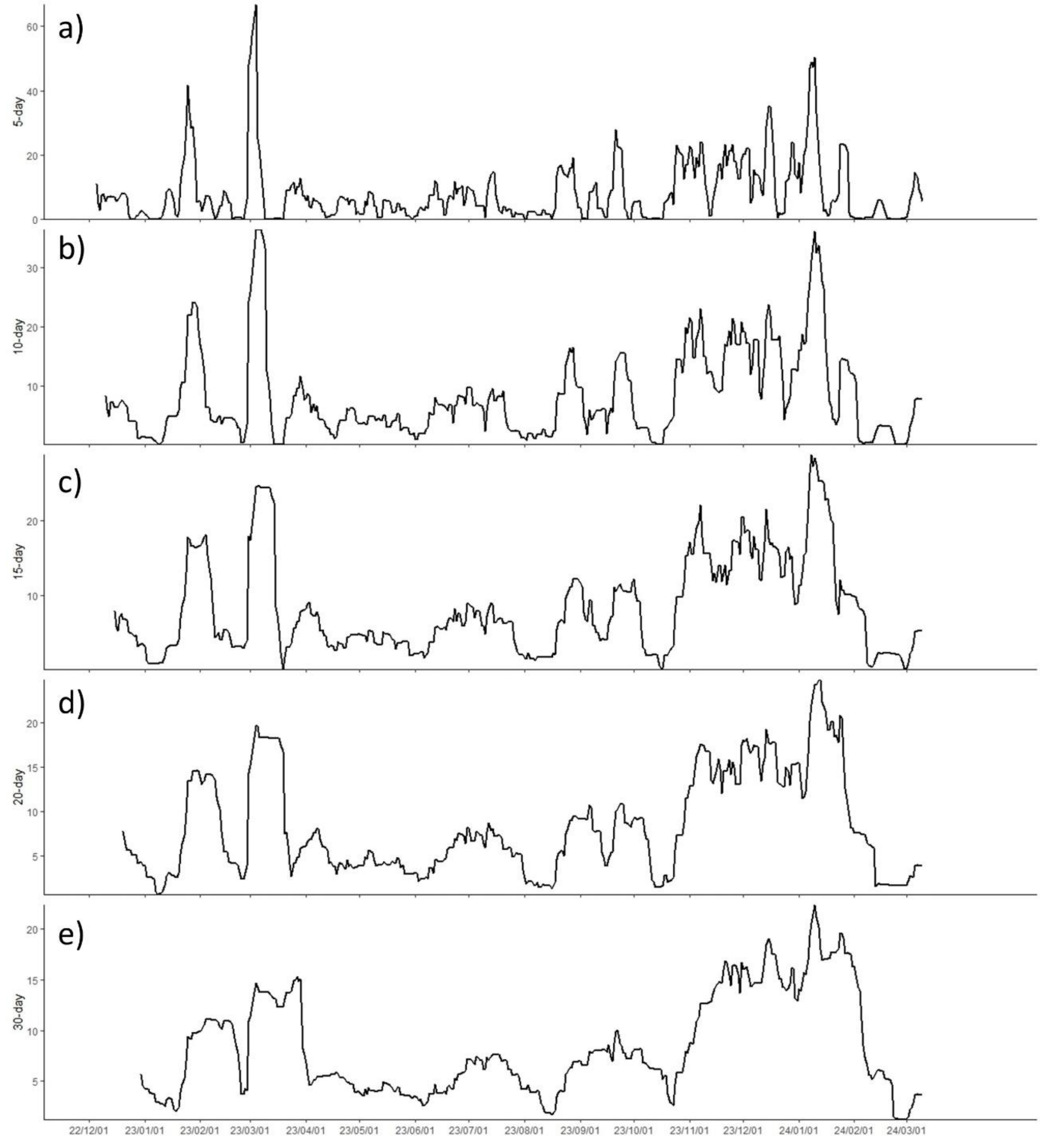

Online Resource 1 a) 5-day, b)10-day, c)15-day, d) 20-day, d) 30-day moving average rainfall.
